# The National Insurance Bill

**Published:** 1911-12-02

**Authors:** 


### THE NATIONAL INSURANCE BILL.

### Our Editor Cross-examined by the King Editor of Weekly Journals.

Many of our confrères of the Press all over the country have been most generous in support of our plea on behalf of the Voluntary Hospitals. It has been necessary for us to deal with many figures, which with the technical questions involved, make a due appreciation of the position sometimes difficult for a layman. It has afforded us great pleasure to have been honoured by the searching scrutiny of our case by one of the ablest and most influential Editors in the country. His main points are embodied in what follows:—

*The King Editor*: I am greatly interested, and desire to help your protest against the National Insurance Bill as it affects Voluntary Hospitals. As a layman I can make very little of the argument in your editorials of the last three issues and the Special Supplement. I cannot see how the Insurance Bill can be represented as creating a fresh class of sick people—75,000 in-patients in London alone. Were none of them ever sick before, or in need of hospital treatment?

*The Editor The Hospital:* All of them have always been liable to need hospital treatment, and have been enabled to obtain it under existing conditions.

*The K. E.*: Then is this class of insured eligible for treatment in Voluntary Hospitals, or not?

*The E. T. H.:* This class was eligible when the applicants were not insured, because they were then bread-winners who, though able to maintain themselves and their families when well, were unable to meet the cost of sickness and treatment for themselves and their families when ill. They belonged to the class which the Voluntary Hospitals were established to help. But when insured they will cease to belong to this class, and Mr. Lloyd George told the deputation of the British Hospitals Association on July 25, 1911, that when the National Insurance Bill passed they should ask the patients: "Are you insured?" "If they were, then they had no right to come to the hospitals."

*The K. E.:* So these former patients of Voluntary Hospitals will not be eligible. Then why worry about need of further accommodation for them, or complain that they will cause a reduction in your subscriptions?

*The E. T. H.:* Because all workmen in the kingdom who have heretofore had the protection of hospital treatment must still be provided with it, or after the passing of this Bill they will be in a worse position, when seriously ill, in regard to medical care than that which the people of any modern nation have ever been called upon to face. The Bill makes no provision for the hospital treatment which insured persons must have, and so Mr. Lloyd George's claim that he "will provide adequate medical treatment for every workman in the kingdom" is baseless. It is therefore essential that the Bill shall provide hospital accommodation for the insured, and we worry about it because we are the only people, apparently, who possess the technical knowledge to enable them to realise the cruelties which must result to British artisans under the Bill if it passes as it stands. Further, there is no provision of the kind included in any of the 35 pages of amendments of which notices have been given on the Report Stage.

*The K. E.:* If the Bill made the insured eligible it would be on the basis that they paid a fair amount for treatment?

*The E. T. H.:* Yes, our proposal is that an agreement should be made by the Insurance Commissioners with each Voluntary Hospital which can devote beds to State cases by which the hospitals will set aside certain pavilions or wards for the insured which will be paid for *pro rata*—*i.e.* the hospitals will thus receive the inclusive actual cost of the State's patients' maintenance, plus 10 per cent. to remunerate the medical practitioners who attend them.

*The K. E.:* I find it impossible to conjecture how you arrive at your statements on page 2 of the Supplement of November 18 of probable reductions in subscriptions.

*The E. T. H.:* You will find the explanation of the figures in The Hospital for July 22, 1911, p. 403, and in The Hospital for October 7, 1911, p. 11. We may explain to you that we have little doubt that the present revenue received from the Hospital Saturday and Sunday Funds, and working-class contributions, will largely, if not entirely, cease after National Insurance is set up, whilst at least half of the revenue derived from annual subscriptions and donations will be lost. In addition to this, there is little doubt, from the communications which have been addressed to Voluntary Hospital managers, and from their evidence, that the large sum at present derived from legacies will much of it be lost, if it does not entirely fail.

*The K. E.:* Again, how do you make out that London Hospitals will lose 43 per cent. of their present income—*i.e.* 23 per cent. in subscriptions and 20 per cent. in legacies?

*The E. T. H.:* The estimate of 23 per cent. is based upon the total ordinary income. Legacies are treated as extraordinary income, and they represent an amount equal to some 25 per cent. of the total ordinary income apart from legacies. If the legacies are added to the total ordinary income then they represent about 20 per cent. of the grand total so arrived at.

*The K. E.*: Still you have not touched my chief difficulties: (1) That the hospitals are to be swamped and ruined by this inrush of new classes of patients; (2) That none of them will obtain relief because they are not eligible. I assure you that your statements are absolutely bewildering to me.

*The E. T. H.*: Let us try and remove your difficulties. It is officially stated that fifteen millions of persons, or 33 per cent. of the population, will be insured under the Bill. Of these the London tion will number 2,500,000 insured. Experience proves that these people will require hospital accommodation to the extent of two hospital beds per thousand, or 5,000 beds at least for their sole use. The London Voluntary Hospitals maintain about 10,500 beds, of which some 10,000 are constantly occupied. If the Bill diminishes the revenue of these hospitals to the extent of something like one-half, as is estimated, it will be difficult, if not impossible, for the managers to continue to maintain more than half the present number available, unless the Bill provides for institutional benefit. Otherwise more than all the beds which are kept open in these Voluntary Hospitals will be required for the treatment of the wives and families of the insured, and a large number of poor persons whom the Bill will not touch. We have shown that the Chancellor himself admits the insured will not be eligible for free medical treatment in a Voluntary Hospital. If the managers of a Voluntary Hospital, with half its revenue destroyed by the Bill, were to take the risk of admitting insured patients to free medical relief and relied upon a generous public to subscribe to a special fund to give them hospital treatment, this would lead to an abuse of the in-patient department probably greater than anything in the way of abuse that has yet occurred in connection with Voluntary Hospitals. Nor is this all, for what person who has to pay for "adequate medical treatment without any taint of charity" for the insured, will be likely to put his hand in his pocket and give a generous donation to provide the same people with hospital care, when he realises that the Government has failed to carry out its undertaking, and has not provided it out of the funds supplied for National Insurance? Further, if a Voluntary Hospital admitted insured persons to free medical relief, it would in all probability lead to the loss of a considerable further portion of any revenue which it might still derive from voluntary sources after the passing of the Insurance Bill.

*The K. E.:* Yes, now I begin to see that we are at the parting of the ways; in fact, it seems to me then that the position created by the Bill as it stands at present is this: It takes away from every workman in the kingdom who becomes insured, adequate medical treatment (which must necessarily include hospital care to be adequate), which he now enjoys. But as an insured person he will not be eligible to receive free medical treatment at all, and yet the Bill provides no means whereby the workman can secure or is supplied with institutional benefit. In other words, the workman cannot, despite Mr. Lloyd George's claim, secure adequate medical treatment under the National Insurance Bill as it stands, for he will be deprived of the hospital treatment which he has heretofore received, without payment, at the Voluntary Hospitals, and there is no other place where he can obtain it.

*The E. T. H.:* That will indeed be the case. It is an amazing fact, but it is true, that the State, under the National Insurance Bill as it stands to-day, with one hand takes toll of the workmen's earnings and their employers' money, to provide the former with adequate medical relief, and with the other deprives them of the privileges they now enjoy in the shape of hospital care, by making them ineligible to receive such care as a free gift at the Voluntary Hospital.

*The K. E.:* You invited my assistance in support of your protest against the National Insurance Bill as it affects the Voluntary Hospitals. It was impossible for me to give that assistance unless you satisfied my unprofessional intelligence with the explanations you have now supplied.

